# Ceftaroline fosamil and treatment of acute bacterial skin and skin structure infections: CAPTURE study experience

**DOI:** 10.1179/1973947813Y.0000000144

**Published:** 2013-12

**Authors:** Paul D Santos, Amanda Davis, Alena Jandourek, Alexander Smith, H David Friedland

**Affiliations:** 1 Clinical Pharmacy, Lakes Region General Hospital, Laconia, NH, USA; 2 Clinical Pharmacy, Presence United Samaritans Medical Center, Danville, IL, USA; 3 Clinical Development, Cerexa, Inc., Oakland, CA, USA; 4 Biostatistics, Cerexa, Inc., Oakland, CA, USA; 5 Clinical Sciences, Cerexa, Inc., Oakland, CA, USA

**Keywords:** ABSSSI, Acute bacterial skin and skin structure infection, CAPTURE, ceftaroline fosamil, Diabetes mellitus, MRSA, Peripheral vascular disease, Obesity

## Abstract

The Clinical Assessment Program and TEFLARO Utilization Registry (CAPTURE) is a multicentre retrospective cohort study in the USA describing treatment of acute bacterial skin and skin structure infection (ABSSSI) with ceftaroline fosamil (CPT-F). Charts for review were chosen by random selection. Among 647 evaluable patients, 52% were obese, 46% had diabetes mellitus (DM), and 19% had peripheral vascular disease (PVD). Methicillin-resistant *Staphylococcus aureus* (MRSA) was recovered in 28% and methicillin-susceptible *S. aureus* (MSSA), 11%. Antibiotics were administered prior to CPT-F treatment in 80%, and concurrently in 39%. Clinical success overall was 85%; in patients with DM, 83%; with PVD, 76%; and in obese patients, 88%. Clinical success was ≧ 79% across all infection types; 81% for MRSA and 83% for MSSA; and 86% for ceftaroline monotherapy and 84% for concurrent therapy. These high clinical success rates support CPT-F as an effective treatment option for ABSSSI, including infections due to MRSA and patients with significant co-morbidities.

## Introduction

Acute bacterial skin and skin structure infections (ABSSSIs) are generally categorized as uncomplicated (e.g. simple abscesses, furuncles, and limited cellulitis) or complicated (e.g. infected ulcers, infected burns, and major abscesses). Complicated ABSSSIs may require hospitalization^1^ and are associated with underlying co-morbidities including diabetes mellitus, peripheral vascular disease, and obesity.

The most common etiologic pathogen in ABSSSI is *Staphylococcus aureus* and methicillin-resistant *S. aureus* (MRSA) accounts for a significant number of these, and some infections may be polymicrobial. In the past decade, increased hospital admissions for these infections in the USA have been largely due to escalating numbers of methicillin-resistant strains of *S. aureus*.^2^^–^4 The incidence of MRSA remains high, although it has recently stabilized.5,^6^ Up to three-quarters of ABSSSIs caused by *S. aureus* are attributed to community-associated MRSA (mainly by the USA 300 strain),^7^^–^10 with fewer cases attributed to strains of health care-associated MRSA or to methicillin-susceptible strains (MSSA).11,^12^

The optimal antistaphylococcal treatment varies by strain because community-associated MRSA, health care-associated MRSA, and MSSA exhibit different antimicrobial susceptibility patterns.7,9,^13^ In clinical practice, however, treatment of ABSSSI is usually empirical and vancomycin remains the drug most commonly used against MRSA. Yet, vancomycin has side effects of nephrotoxicity and ototoxicity, and reduced susceptibility to this agent has been identified in the USA and elsewhere.14,^15^ Newer alternatives to vancomycin are linezolid and daptomycin; both are active against *S. aureus*, but lack activity against Gram-negative pathogens that may also be implicated in polymicrobial ABSSSI.16,^17^ Tigecycline, another new agent indicated for the treatment of ABSSSIs, has broad spectrum antibacterial activity, but has been associated with the potentially serious side effects of pancreatitis and Stevens–Johnson syndrome, and increased mortality.^18^^–^22

Ceftaroline fosamil for injection (TEFLARO; Forest Laboratories., Inc., Jersey City, NJ, USA) is a cephalosporin with broad spectrum activity including activity against MRSA and against many pathogens that cause ABSSSIs. It has been approved by the FDA for the indication of ABSSSI caused by susceptible isolates of *S. aureus* (both MRSA and MSSA), *Streptococcus pyogenes*, *S. agalactiae*, *Escherichia coli*, *Klebsiella pneumoniae*, and *K. oxytoca*. Ceftaroline has activity against common aerobic community-acquired Gram-negative organisms, although it is not active against strains that are AmpC-derepressed or produce extended-spectrum β-lactamases.23,^24^ Besides MRSA, ceftaroline has in vitro activity against many drug-resistant pathogens, including vancomycin-resistant *S. aureus* and macrolide-resistant β-hemolytic streptococci.^24^^–^26

Ceftaroline has a bactericidal mechanism of action like other members of the β-lactam family, and prevents bacterial cell wall biosynthesis through irreversible binding to penicillin-binding proteins (PBPs), including PBP2a in MRSA.^27^ The spectrum of activity and mechanism of action support the use of ceftaroline in ABSSSIs, particularly in infections caused by emerging resistant and highly virulent pathogens such as MRSA, including the USA 300 strain.^24^

The Clinical Assessment Program and TEFLARO Utilization Registry (CAPTURE) is a retrospective cohort study designed to collect information on the clinical use of ceftaroline fosamil for ABSSSI and community-acquired bacterial pneumonia in the USA. Data were collected by review of medical charts. The study experience for the treatment of ABSSSI from the first year, August 2011 through August 2012, is presented here.

## Methods

### Study design

This is a multicentre, retrospective cohort study of adult patients treated with intravenous ceftaroline fosamil for ABSSSI. The study was approved by each institution’s ethics committee. The analyses presented here include data collected from August 2011 to August 2012. Data collection was by retrospective review of randomly ordered patient charts identified from pharmacy listings in centres across the USA. To ensure retrospective collection of data, patients had to have received their final dose of ceftaroline at least 30 days before the start of data collection. Patients who received at least two consecutive doses of ceftaroline for ABSSSI were included.

### Study population

Included in the study were adult (≧ 18 years) patients diagnosed with ABSSSI, defined as a skin or skin structure infection involving deeper soft tissue or requiring significant surgical intervention. Examples are a wound infection (surgical or traumatic), a major abscess, an infected ulcer, or deep and extensive cellulitis. Patients were excluded if information on their ceftaroline fosamil dosing or discharge from hospital was missing from their patient charts, or if data relating to ceftaroline fosamil dosing had previously been extracted from their charts for this study.

### Data collection

For each eligible patient, data relating to demographics, medical, and surgical histories, clinical signs, and symptoms at the time of diagnosis and at the end of ceftaroline treatment, and ABSSSI pathogens were collected. Other antimicrobial agents administered for ABSSSI before or during ceftaroline treatment were recorded, as were the location of care (intensive care unit [ICU], hospital ward, outpatient parenteral antibiotic therapy units) and the destination of the patient following discharge from the hospital. Reasons for discontinuation of ceftaroline fosamil including those due to adverse events were recorded.

### Statistical analyses

Statistical analyses were performed on the data using SAS Version 9.2. The data were summarized using primarily descriptive statistics, based on two analysis sets: the enrolled population and the evaluable population. The enrolled population included all patients meeting inclusion/exclusion criteria, while the evaluable population included all patients who met all eligibility criteria and had a clinical outcome of clinical success or clinical failure. Clinical success was defined as either (1) clinical cure with no further need for antibiotic, or (2) clinical improvement with switch to oral antibiotic, or (3) in some cases, review of information including queries confirming that a patient was improving on treatment without evidence of failure at the time of ceftaroline fosamil discontinuation.

Clinical outcome was analysed according to ABSSSI type, antibiotic usage (ceftaroline as monotherapy or concurrent therapy, and as first-line therapy (no prior antibiotics) or second-line therapy), and isolation of *S. aureus* (both methicillin-resistant and -susceptible). Clinical outcome was also analysed for subsets of patients considered to be at special risk, that is, the obese (BMI ≧ 30 kg/m^2^) and those with diabetes mellitus or peripheral vascular disease.

## Results

### Patient characteristics

Data were collected from 33 study centres for a total of 694 enrolled patients with ABSSSIs. The enrolled population had near identical baseline characteristics to the evaluable population and data for the evaluable population are presented.

The evaluable population included 647 patients and the demographics data are shown in Table 1. The mean age of the patients was 58·0 years (range: 18·0–106·0 years), the mean weight was 97·5 kg (52% were obese and 21% were overweight), and 55% of patients were male. Co-morbidities included diabetes mellitus in 46% and peripheral vascular disease in 19% of patients. At the start of ceftaroline fosamil treatment, the majority (92%) of the patients were treated in general hospital wards, 7% were in ICUs, and 1% received outpatient parenteral antibiotic therapy or home intravenous therapy. Most patients (77%) were subsequently discharged home and 22% were transferred to another care facility; discharge data were missing for 1% of patients.

**Table 1:** Baseline characteristics — evaluable patients (*N*  =  647)

Gender	Male, *n* (%)	359 (55·5)
	Female, *n* (%)	288 (44·5)
Age at baseline, years	Mean (SD)	58·0 (18·1)
	Median (range)	59·0 (18·0–106·0)
Age group	< 65 years, *n* (%)	406 (62·8)
	≧ 65 years, *n* (%)	241 (37·2)
Weight, kg	Mean (SD)	97·5 (34·0)
(*n * = 629)	Median (range)	90·3 (40·9–289·7)
BMI, kg/m^2^	Mean (SD)	33·6 (11·2)
(*n * = 605)	Median (range)	31·2 (15·3–91·6)
BMI group	Underweight (< 18·5 kg/m^2^), *n* (%)	10 (1·5)
	Normal (18·5–< 25 kg/m^2^), *n* (%)	126 (19·5)
	Overweight (25·0–< 30 kg/m^2^), *n* (%)	134 (20·7)
	Obese (≧ 30 kg/m^2^), *n* (%)	335 (51·8)
Relevant medical condition	Any surgical or medical history, *n* (%)	499 (77·1)
	Diabetes mellitus, *n* (%)	295 (45·6)
	Peripheral vascular disease, *n* (%)	120 (18·5)

### Disease characteristics

The mean and median length of ABSSSI diagnosis before treatment with ceftaroline fosamil in the evaluable population were 9·3 and 2·0 days (range: 0·0–475·0 days). Some patients presented with more than one type of ABSSSI. The most common infection type was deep/extensive cellulitis (47% of patients), followed by major abscess (19%), infected ulcer (18%), infected surgical wound (15%), SSSIs (and no other infections noted) in diabetes mellitus/peripheral vascular disease (DM/PVD) patients (13%), and infected traumatic wound (7%). Other infection types (erysipelas, infected animal bite, infected burn, and other infected site) were each reported in < 5% of patients. Infection was present at more than one body site in some patients. The lower limbs were the most common sites of infection, with the leg/thigh infected in 42% of patients and the foot in 27%. Infection on the arm/forearm, abdomen, buttocks, head/neck and hand occurred in 5–9% of patients; a small proportion of patients (< 5%) had sites of infection on the chest, groin, and back.

Clinical signs and symptoms of erythema, tenderness, and swelling were present in 80%, 68%, and 65% of patients, respectively, at the time of diagnosis of the ABSSSI; other protocol-defined signs and symptoms were discharge (35%), warmth (32%) and fluctuance (9%). At the end of ceftaroline treatment, these rates were reduced two- to five-fold, to erythema, 33% of patients; tenderness, 24%; swelling, 20%, discharge (10%), warmth (6%), and fluctuance (2%).

### Antibiotic usage

Patients were treated with ceftaroline fosamil for a mean (± SD) duration of 6·1 (± 5·5) days and patients received a mean (± SD) of 10·6 (± 11·0) doses of ceftaroline fosamil. Obese patients (BMI ≧ 30 kg/m^2^) received ceftaroline fosamil for a similar duration of therapy [mean (± SD): 5·9 (± 5·5) days] as patients with normal BMI [mean (± SD), 6·1 (± 5·6) days]. Ceftaroline fosamil was administered as first-line therapy in 20% of patients. The remaining patients received prior antibiotics, most commonly glycopeptides (44%), penicillins (25%), other cephalosporins (22%), and lincosamides (17%); sulfonamides, quinolones, and oxazolidinones were administered to 5–10% of patients. The majority of patients (61%) received ceftaroline as monotherapy: 88 (14%) as initial therapy and 305 (47%) as switch therapy. In the patients who were on ceftaroline fosamil concurrently with other antibiotics (39%), the most frequently used concurrent antibiotics were lincosamides (12% of evaluable patients) and glycopeptides (9%) (Table 2).

**Table 2:** Ceftaroline fosamil usage — evaluable patients (*N * =  647)

		*n* (%)
Ceftaroline as first-line therapy	All patientsMonotherapy	132 (20·4)88 (13·6)
	Concurrent therapy	44 (6·8)
Prior antibiotics (with ≧5% of patients)	All antibioticsGlycopeptidesPenicillins	515 (79·6)284 (43·9)163 (25·2)
	Cephalosporins	141 (21·8)
	Lincosamides	110 (17·0)
	Sulfonamides	54 (8·3)
	Quinolones	38 (5·9)
	Oxazolidinones	34 (5·3)
Ceftaroline monotherapy	All patientsFirst-line therapySecond-line therapy	393 (60·7)88 (13·6)305 (47·1)
Concurrent therapy (with ≧5% of patients)	All antibioticsLincosamidesGlycopeptides	254 (39·3)76 (11·7)55 (8·5)

### Clinical success rates

The overall clinical success rate was 85% (550/647) and rates were high across all categories examined. Clinical success rates across infection types were: 85% (259/305) for deep/extensive cellulitis, 87% (109/125) for major abscesses, 79% (92/117) for infected ulcers, 81% (78/96) for infected surgical wounds, 89% (39/44) for infected traumatic wounds, and 86% (74/86) for SSSIs (and no other infections noted) in DM/PVD patients. Clinical success rates were 83% (244/295) among patients with diabetes mellitus, 76% (91/120) among those with peripheral vascular disease, and 87% (274/314) among those with no history of either. The clinical success rate in elderly patients (≧ 65 years) was 82% (197/241) and in younger patients (< 65 years), 87% (353/406). In patients in the obese group, it was 88% (294/335); in the overweight group, 83% (111/134); and in the underweight/normal group, 84% (114/136).

Clinical success rates did not vary markedly with prior or concurrent antibiotic usage. As first-line therapy, ceftaroline had a success rate of 81% (71/88) when administered as monotherapy and 82% (36/44) when administered concurrently with other antibiotics. Where prior antibiotics were used, the clinical success rate was 87% (266/305) among patients who were switched to ceftaroline monotherapy and 84% (177/210) among those who received ceftaroline concurrently with other antibiotics. In the subset of 284 patients who were administered glycopeptides initially, the clinical success rate with second-line ceftaroline therapy was 88%. Ceftaroline monotherapy gave a success rate of 86% (337/393), while concurrent use with other antibiotics gave 84% (213/254) success. Among those patients who received glycopeptides as concurrent therapy, the clinical success rate was 85% (47/55).

A small proportion of patients (2%) discontinued ceftaroline treatment as a consequence of adverse events.

### Pathogens isolated

One or more ABSSSI pathogens were isolated from 363 (56%) patients. Among these, 191 isolates of MRSA and 74 isolates of MSSA were cultured from 178 (28%) and 70 (11%) patients, respectively. The main source for these isolates in both cases was skin/soft tissue/wound cultures (93% and 88%, respectively); the remaining isolates were cultured from blood. Clinical success rates were 81% (293/363) among patients with any pathogens isolated and 90% (257/284) among those without; and 81% (144/178) and 83% (58/70) among patients with MRSA and MSSA, respectively.

## Discussion

These data on the contemporary use of ceftaroline fosamil in the treatment of ABSSSIs from the first year of the CAPTURE study show that ceftaroline is being used in patients with significant underlying co-morbidities and in those with severe disease. Additionally, ceftaroline is often being used as second-line treatment after other antibiotics have been administered.

ABSSSIs can occur on any part of the body but most frequently occur on the lower extremities,^28^ and the infection site and infection type distribution is reflected in this analysis. The majority of patients experienced at least one clinical sign or symptom of ABSSSI at the start of treatment with ceftaroline fosamil; however, the incidence of each sign and symptom decreased markedly by the end of treatment, suggestive of the effectiveness of treatment.

Ceftaroline was effective as monotherapy, whether as first-line therapy (81% clinical success) or as second-line therapy (87%). Among the patients who received first-line therapy with antibiotics other than ceftaroline, approximately half had received glycopeptides, which may suggest inadequate or delayed clinical response to glycopeptide therapy. A favourable clinical response in these patients was seen after the addition of or switch to ceftaroline treatment (88%).

*In vitro* studies have shown the high activity of ceftaroline against *S. aureus*, and MIC_90_ (MIC range) values of 0·25 (≤ 0·08–1) μg/ml for MSSA and 1 (0·12–2) μg/ml for MRSA are commonly reported.23,^24^ No MIC data were available for the isolates included in this analysis of the CAPTURE study as commercial testing kits were not available at the time; however, ceftaroline was effective against the *S. aureus* isolated, irrespective of methicillin resistance, with clinical success rates of 81–83%. Although the most commonly isolated pathogen was *S. aureus*, a range of other pathogens (e.g. *S. agalactiae* and *E. coli*) were each isolated from < 20 patients. In infections such as ABSSSIs where, in some circumstances, more than one causative pathogen may be implicated, the broad spectrum activity of ceftaroline would offer the advantage of its use as monotherapy.

As a retrospective cohort study, CAPTURE is designed to assess the effectiveness of ceftaroline in clinical practice. The overall clinical success rate was 85% and the majority of the patients were discharged home. These findings compare favourably with the results from two double-blinded Phase III clinical studies (each with 700 patients) comparing ceftaroline fosamil with vancomycin±aztreonam in complicated ABSSSIs. An integrated analysis of the two studies showed comparable clinical cure rates of 92–95% for ceftaroline fosamil and the comparator.^29^ The overall success rate of 85% in CAPTURE is lower than those in the clinical studies and reflect differences in the study populations. Compared with patients included in the clinical studies, more CAPTURE patients were already hospitalized at study entry (92% versus 78%), aged ≧ 65 years (37% versus 26%), and had a higher rate of co-morbidities (obesity: 52% versus 32%; diabetes mellitus: 46% versus 18%; peripheral vascular disease: 19% versus 13%). Additionally, use of prior antibiotics was extensive in CAPTURE, but restricted in the clinical studies.

In both clinical studies, ceftaroline was well tolerated and demonstrated a safety profile reflective of the cephalosporin class.^30^ In CAPTURE, an adverse event reported to be the reason for discontinuation of ceftaroline fosamil was reported in few patients.

As CAPTURE is a retrospective chart review study, it has the limitations inherent to this study design. Also, this is not a comparative study which limits inferences that can be made to other antibiotic agents, and the population in CAPTURE is different in many respects (i.e. obesity, co-morbidities, second-line therapy) than was seen in the Phase III studies of ceftaroline fosamil. Additional limitations of the study include the absence of data on the isolates’ susceptibility to ceftaroline and sparse collection of information on treatment-limiting adverse events. The ABSSSI data from the first year of the CAPTURE study provide information on the contemporary clinical use of ceftaroline fosamil.

### Summary

High clinical success rates were seen with contemporary clinical use of ceftaroline fosamil for the treatment of ABSSSIs, including infections in patients with significant co-morbidities such as diabetes mellitus, peripheral vascular disease, and obesity. The clinical response was favourable in patients who were treated with ceftaroline fosamil as first-line therapy and in those who were treated with ceftaroline fosamil as second-line therapy. Ceftaroline demonstrated high clinical success for infections associated with either MRSA or MSSA. Ceftaroline has a safety profile reflective of the cephalosporin class and discontinuation of treatment due to adverse events was reported in few patients.

These data from the CAPTURE study support the use of ceftaroline fosamil for the treatment of ABSSSIs.
